# Complete mitochondrial genome of the ambush bug, *Amblythyreus gestroi* (Hemiptera: Reduviidae)

**DOI:** 10.1080/23802359.2018.1511854

**Published:** 2018-10-27

**Authors:** Zhuo Chen, Fan Song, Wanzhi Cai

**Affiliations:** Department of Entomology and MOA Key Lab of Pest Monitoring and Green Management, College of Plant Protection, China Agricultural University, Beijing, China

**Keywords:** Mitochondrial genome, reduviidae, phymatinae, *Amblythyreus gestroi*

## Abstract

The mitochondrial genome (mitogenome) of *Amblythyreus gestroi* is described in the present paper. The complete mitogenome is a 15,228 bp circular DNA molecule, containing 13 protein-coding genes, 22 tRNA genes, two rRNA genes and a control region. Genome organization, nucleotide composition and codon usage of the mitogenome are noted, secondary structures of all tRNAs are predicted. The monophyly of Phymatinae is highly supported by the phylogenetic tree. Phylogenetic implications of the *A. gestroi* mitogenome is briefly discussed.

The ambush bug genus *Amblythyreus* Westwood, 1837 (Hemiptera: Heteroptera: Reduviidae) was firstly described as a subgenus of *Macrocephalus* (Westwood 1837) and then upgraded to the generic level (Amyot and Serville 1843). This genus currently contains 15 species, occurring in the Oriental Region exclusively (Froeschner and Kormilev 1989). Handlirsch (1897) described a Myanmar species named *Amblythyreus gestroi*, this species is also recorded from China and India (Hsiao and Liu 1981; Putshkov and Putshkov 1996). In this study, the complete mitochondrial genome (mitogenome) of *A. gestroi* is sequenced and described. Voucher specimen (No. VCim-00114) was deposited in the Entomological Museum of China Agricultural University, Beijing, China. The sequence was deposited in GenBank under the accession number KY069956.

The complete mitogenome of *A. gestroi* is a typical circular DNA of 15, 228 bp, including 37 genes (13 protein-coding genes, 22 tRNA genes, two rRNA genes) and a control region. Gene order is identical to the putative ancestral arrangement of insects (Cameron 2014; Song et al. 2016), no rearrangement occur in this mitogenome. Totally, 58 bp overlapped nucleotides between neighbouring genes in 13 locations, ranging from 1 to 17 bp in size.

The nucleotide composition of this mitogenome is significantly biased towards A + T (72.9%) with positive AT-skew (0.11) and negative CG-skew (−0.17). All of 13 protein-coding genes initiate with ATN as the start codon (six with ATA, five with ATG, and two with ATT). The stop codon TAA and TAG are assigned to seven and three protein-coding genes, respectively, whereas a single T residue is used by *COIII*, *ND3* and *ND5* as incomplete stop codon which is commonly in Heteroptera mitogenomes (Li et al. 2012; Shi et al. 2012).

The length of tRNA genes range from 61 to 70 bp. All tRNA genes can be folded into the typical clover-leaf secondary structure except for *tRNA^Ser(AGN)^*, in which its DHU arm simply formed a loop, as is the case with most other insects (Li et al. 2012; Shi et al. 2012). The *lrRNA* is 1244 bp in length with an A + T content of 75.2%, and the *srRNA* is 759 bp long with an A + T content of 74.3%. The 691 bp control region is located between *srRNA* and *tRNA^Ile^* and shows significantly AT bias (65.6%). Except for the control region, there are other nine inter-genic regions range from 1 ∼ 45 bp in size, of which the longest one is located between *tRNA^Ile^* and *tRNA^Gln^*.

Phylogenetic tree was constructed by maximum-likelihood (ML) method (Figure 1). This phylogenetic analysis confirms the monophyly of Phymatinae, which is congruent with previous hypotheses (Weirauch et al. 2011; Masonick et al. 2017). The sister relationship between *A. gestroi* and *Carcinocoris binghami* is highly supported, this result may imply the close relationship of Macrocephalini and Carcinocorini. *Amblythyreus* belongs to the most speciose phymatine tribe Macrocephalini, which is suspected to be a paraphyly group (Masonick et al. 2017), the mitogenomic information of *A. gestroi* provides a basic data for future research investigating the unclear relationships within Macrocephalini.

**Figure 1.**
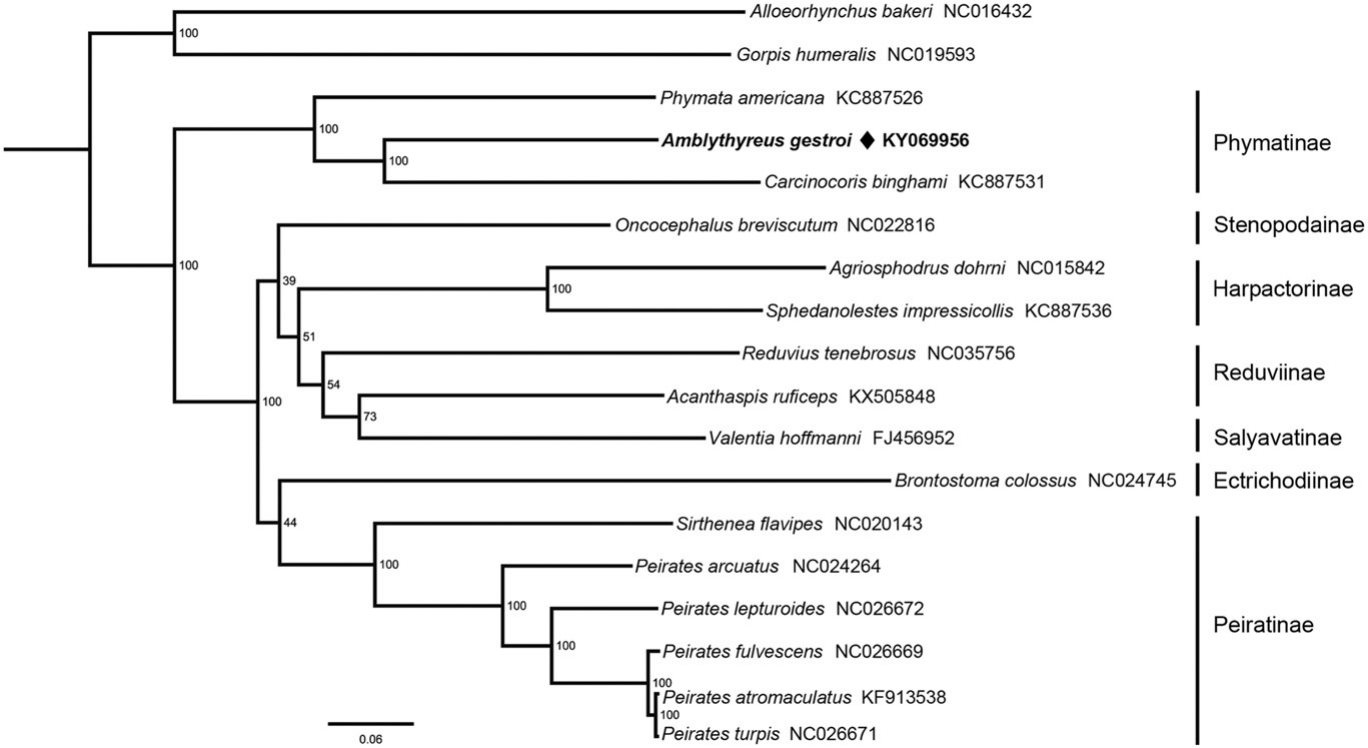
Phylogenetic relationship of 16 Reduviidae species inferred from ML analysis based on 13 protein-coding genes and two rRNA genes (12,697 bp). Phylogenetic tree was generated by IQ-TREE 1.6.5 (Trifinopoulos et al. 2016) under the GTR + I + G model. The nodal values indicate the bootstrap percentages obtained with 1000 replicates. Alphanumeric terms indicate the GenBank accession numbers.
